# Optimization of cellulolytic enzyme components through engineering *Trichoderma reesei* and on-site fermentation using the soluble inducer for cellulosic ethanol production from corn stover

**DOI:** 10.1186/s13068-018-1048-5

**Published:** 2018-02-23

**Authors:** Yong-Hao Li, Xiao-Yue Zhang, Fei Zhang, Liang-Cai Peng, Da-Bing Zhang, Akihiko Kondo, Feng-Wu Bai, Xin-Qing Zhao

**Affiliations:** 1 State Key Laboratory of Microbial Metabolism, Joint International Research Laboratory of Metabolic and Developmental Sciences, and School of Life Sciences and Biotechnology, Shanghai Jiao Tong University, Shanghai, 200240 China; 2 School of Life Science and Biotechnology, Dalian University of Technology, Dalian, 116023 China; 3 Department of Chemical Science and Engineering, Graduate School of Engineering, Kobe University, Kobe, 657-8501 Japan; 4 Present Address: School of Chemistry and Chemical Engineering, Chongqing University of Science and Technology, Chongqing, 401331 China

**Keywords:** *Trichoderma reesei*, On-site cellulase production, Soluble inducer, β-Glucosidase, Lignocellulosic ethanol

## Abstract

**Background:**

Cellulolytic enzymes produced by *Trichoderma reesei* are widely studied for biomass bioconversion, and enzymatic components vary depending on different inducers. In our previous studies, a mixture of glucose and disaccharide (MGD) was developed and used to induce cellulase production. However, the enzymatic profile induced by MGD is still not defined, and further optimization of the enzyme cocktail is also required for efficient ethanol production from lignocellulosic biomass.

**Results:**

In this study, cellulolytic enzymes produced by *T. reesei* Rut C30 using MGD and alkali-pretreated corn stover (APCS) as inducer were compared. Cellular secretome in response to each inducer was analyzed, which revealed a similar enzyme profile. However, significant difference in the content of cellulases and xylanase was detected. Although MGD induction enhanced β-glucosidase production, its activity was still not sufficient for biomass hydrolysis. To overcome such a disadvantage, *aabgl1* encoding β-glucosidase in *Aspergillus aculeatus* was heterologously expressed in *T. reesei* Rut C30 under the control of the *pdc1* promoter. The recombinant *T. reesei* PB-3 strain showed an improved β-glucosidase activity of 310 CBU/mL in the fed-batch fermentation, 71-folds higher than that produced by the parent strain. Meanwhile, cellulase activity of 50 FPU/mL was detected. Subsequently, the crude enzyme was applied for hydrolyzing corn stover with a solid loading of 20% through separate hydrolysis and fermentation (SHF) and simultaneous saccharification and fermentation, respectively, for ethanol production. Better performance was observed in the SHF process, through which a total of 119.9 g/L glucose was released within 12 h for concomitant ethanol production of 54.2 g/L.

**Conclusions:**

The similar profile of cellulolytic enzymes was detected under the induction of MGD and APCS, but higher amount of cellulases was present in the crude enzyme induced by MGD. However, β-glucosidase activity induced by MGD was not sufficient for hydrolyzing lignocellulosic biomass. High titers of cellulases and β-glucosidase were achieved simultaneously by heterologous expression of *aabgl1* in *T. reesei* and fed-batch fermentation through feeding MGD. We demonstrated that on-site cellulase production by *T. reesei* PB-3 has a potential for efficient biomass saccharification and ethanol production from lignocellulosic biomass.

**Electronic supplementary material:**

The online version of this article (10.1186/s13068-018-1048-5) contains supplementary material, which is available to authorized users.

## Background

Lignocellulosic biomass has attracted great attention for producing biofuels and chemicals due to its abundance and renewability [1]. However, the high cost of cellulase production is the major bottleneck for the economic feasibility [2]. *Trichoderma reesei* has been widely studied owing to its capacity to secrete large amounts of proteins. Among various *T. reesei* strains, the well-known hyper-cellulase producer *T. reesei* Rut C30 is of particular interest for the industrial production of cellulases [3].

Cellulase production by *T. reesei* requires induction. Cellulolytic enzyme cocktails from *T. reesei* with different compositions were obtained when different inducers were used, which lead to different biomass hydrolysis efficiency [4]. Usually, lignocellulose is a natural inducer for cellulase production by *T. reesei*. However, the low titer and productivity of cellulases remain as challenges [5]. We previously developed a low-cost soluble inducer (mixture of glucose and disaccharide, MGD), which enabled high cellulase titers and productivity [6]. However, the profile of cellulolytic enzymes induced by MGD has not been investigated in detail.

The cellulolytic enzyme system of *T. reesei* consists of at least four types of enzymes: cellulases, hemicellulases, ligninases, and pectinases [7], and cellulases contain three major enzyme components: (1) endoglucanases that randomly cleave glycosidic linkages; (2) cellobiohydrolases that yield cellobiose through hydrolyzing glycosidic linkages from the ends of cellulose molecule; and (3) β-glucosidases which convert cellobiose into glucose [8]. *T. reesei* produces at least two cellobiohydrolases (CEL7A and CEL6A), five endoglucanases (CEL7B, CEL5A, CEL5B, CEL12A, CEL45A), two characterized β-glucosidases (CEL3A and CEL1A), and additional five predicted β-glucosidases (CEL3B, CEL3D, CEL1B, CEL3C, CEL3E) [9]. In addition, synergistic proteins have also been shown to play important roles in the oxidative degradation of cellulose. These synergistic proteins include the expansin-like protein swollenin (SWOI) and lytic polysaccharide monooxygenases (LPMOs), of which was recently reclassified from family GH61 to family AA9 (Auxiliary family activity 9) [9].

*T. reesei* cellulases normally have low β-glucosidase activity (BGA), which causes the accumulation of cellobiose that resulted in the inhibition of the production of endo- and exo-cellulases [10, 11]. In our previous study, improved β-glucosidase production of *T. reesei* Rut C30 engineered by an artificial transcription factor leads to improved glucose release [11]. Increased BGA was also achieved by overexpression of either heterologous or endogenous β-glucosidase genes using the cellulase or xylanase promoters, but unfortunately, reduced expression of cellulase or xylanase genes was observed [12–14]. Employment of strong constitutive promoter for *bgl1* gene overexpression in combination with the feeding of soluble inducer [6] may be an alternative strategy for improving BGA without comprising the production of other cellulolytic enzymes. However, such attempt has not been reported so far.

In this study, the profile of cellulolytic enzymes induced by MGD was investigated in detail. In addition, to further improve BGA in *T. reesei*, gene (*aabgl1*) encoding β-glucosidase 1 in *Aspergillus aculeatus* was overexpressed in *T. reesei* Rut C30 under the constitutive pyruvate decarboxylase promoter *pdc1*. The recombinant strain was used for cellulase production by fed-batch fermentation through feeding the soluble inducer. Furthermore, cellulosic ethanol production was performed by *Saccharomyces cerevisiae* through separate hydrolysis and fermentation (SHF) or simultaneous saccharification and fermentation (SSF) using the crude cellulases. To our best knowledge, this is the first time that high titers of both cellulases and β-glucosidase have been achieved by the fed-batch fermentation. The lignocellulolytic enzyme cocktail reported in this study showed a great advantage in the saving cost for the supplementation of commercial β-glucosidase and facilitate cellulosic biomass bioconversion.

## Methods

### Microorganisms and culture media

The *T. reesei* Rut C30 strain was purchased from the ARS (NRRL) Culture Collection, and the seed and fermentation medium for *T. reesei* Rut C30 in shake flask were described elsewhere [6]. *Escherichia coli* DH5α was used as host strain to construct various plasmids. *Agrobacterium tumefaciens* AGL-1 was used for *A. tumefaciens*-mediated transformation (AMT) of *T. reesei*. *E. coli* and *A. tumefaciens* were cultured on LB (Luria–Bertani) medium. The commercial Angel Yeast *Saccharomyces cerevisiae* (Angel Yeast Co., Hubei, China) was used for cellulosic ethanol fermentation. MGD and alkali pretreatment corn stover (APCS) were prepared as described in our previous study [6].

### Preparation of two kinds of inducers

The soluble inducer MGD was prepared as described previously, which was produced from 600 g/L glucose transglycosylated by β-glucosidase [6]. Another inducer is pretreated corn stover, which was produced from corn stover pretreated by 2% NaOH for 90 min [15]. The chemical compositions of APCS were determined to be 62.6% cellulose, 21.4% hemicellulose, and 8.2% lignin of the dry mass (DM).

### Cellulase and β-glucosidase production by fed-batch fermentation

The cultivation medium and conditions for seed and hyphal growth were the same as described in our previous study [6]. A 7 L fermenter was used for cellulase and/or β-glucosidase production with the initial volume of 3 L. When MGD was used as inducer for cellulase and/or β-glucosidase production, the media and culture conditions were consistent with our previous reports [6]. When APCS was used for cellulase production, the fermentation conditions were the same with that of MGD except that the APCS was fed at 8 h intervals at a rate of 0.8 g/L/h [16].

### Component analysis of the lignocellulose cocktails by different inducers

Samples taken from fermenters were centrifuged at 6000 rpm for 5 min at 4 °C, the supernatant was collected and filtrated through a 0.2 μm filter, then it was concentrated using a lyophilizer, and the protein concentration was measured by the Bradford method (Sangong, China). Totally 100 μg protein for each sample was mixed with fivefold volume of cold acetone at − 20 °C for 1 h, then the protein solution was centrifuged at 12,000 rpm for 15 min. The precipitate was collected, and incubated at 60 °C for 1 h, and then 50 μL dissolution buffer and 4 μL reducing reagent were added in the precipitate. The 2 μL cysteine-blocking reagent was added into the protein solution at room temperature for 10 min, followed by the centrifugation with 10 Da ultrafiltration tube at 12,000 rpm for 20 min. Then the ultrafiltration tubes were purged by 100 μL dissolution buffer and centrifuged at 12,000 rpm for 1 min for three times. Protein digestion was performed by adding 50 μL sequencing-grade trypsin (50 ng/μL) to an ultrafiltration tube and incubated at 37 °C for 12 h. After ultrafiltration tube was centrifuged at 12,000 rpm for 20 min, the filtrate with the peptides was gathered for protein labeling and MS analysis.

The iTRAQ (isobaric tags for relative and absolute quantification) labeling of peptides obtained from the test samples induced by MGD or APCS was performed using an iTRAQ reagent Multiplex kit (Applied Biosystems, Foster City, CA) according to the manufacturer’s protocol. The LC–MS/MS analysis of fractionated labeled sample was performed with a QStar Elite mass spectrometer (Applied Biosystems/MDS Sciex) coupled with online microflow HPLC system (Shimadzu). The peak areas of the iTRAQ reporter ions reflect the relative abundance of the proteins in the samples. Sequences were mapped based on the reference genome of *T. reesei* Rut C30, obtained from the Joint Genome Institute (JGI) Genome Portal (http://genome.jgi.doe.gov/pages/search-for-genes.jsf?organism=TrireRUTC30_1).

### Construction of the *aabgl1* expression cassette

The primers used in this study are listed in Additional file 1: Table S1. The *pdc1* promoter (1500 bp) and *pdc1* terminator (1000 bp) were amplified from *T. reesei* Rut C30 genome by PCR with the corresponding primers. Similarly, *aabgl1* gene (GenBank: D64088.1) and hygromycin B cassette were amplified from *S. cerevisiae* MNII/cocδBEC3 genome [17] and pAN7-1 (GenBank: Z32698.1), respectively. The resistance gene of hygromycin B cassette was inserted into pCB301 (GenBank: AF139061.1) digested by *Spe*I, yielding the backbone vector pCBHYGB. The amplified *pdc1* promoter, *aabgl1* gene, and *pdc1* terminator were fused to pCBHYGB using the seamless cloning master mix according to the manufacturer’s instructions (Sangon Biotech, Shanghai, China), generating the vector pCBHYGB-PB (Additional file 3: Figure S1A) which was verified by sequencing.

### Transformation of *T. reesei* Rut C30 and qPCR analysis

The vector pCBHYGB-PB harboring the overexpression cassette of *aabgl1* was transformed into *T. reesei* Rut C30 using *A. tumefaciens*-mediated transformation (ATMT) method previously developed [18]. Twenty transformants were verified by PCR with the primer aabgl1-F/R and evaluated in shake flasks. qPCR analysis of the transformant *T. reesei* PB-3 was carried out according to the previous work [6], and the primers are listed in Additional file 1: Table S1. Transcription level of *sar1* was used as an internal control.

### Determination of copy numbers by qPCR analysis

The genomic DNA of *T. reesei* transformants, isolated using fungal DNA extraction kit (Sangon Biotech, Shanghai, China), was used as template for qPCR analysis. The *aabgl1* copy number in transformants was determined as described by Mason et al. [19]. The *tef1α* (translation elongation factor 1-alpha, NCBI Reference Sequence: XM_006963994.1) gene was used to represent a single copy gene [14].

### Enzymatic saccharification in shake flasks

The crude cellulases induced by different inducers from *T. reesei* Rut C30 were collected by centrifugation (6000 rpm, 5 min). The enzymes were mixed with APCS and then re-suspended in HAc–NaAc buffer (0.2 M and pH 4.8) with an enzyme loading of 3 mg protein or 20 FPU/g APCS and a solid loading of 5% (w/v). Reaction solutions were incubated in a shaking bath at 100 rpm, 50 °C. The samples were collected at different time points, followed by centrifugation at 8000 rpm for 2 min, and the supernatants were subjected to analysis of glucose concentration.

### Ethanol fermentation in bioreactor using in-house cellulases

SHF and SSF processes were carried out in 2.5 L fermenter (Kobio Tech Co., Korea) at a total biomass loading of 20% DM (200 g solid/L H_2_O). The pH value was maintained at 4.8 by adding 0.2 M H_2_SO_4_ or aqueous ammonia automatically. Angel yeast (1 g/L) was used for ethanol fermentation containing 1 g/L yeast extract and 1 g/L peptone. In SHF process, APCS was degraded by the recombinant *T. reesei* PB-3 crude enzyme with enzyme loading of 30 FPU/g biomass at 50 °C for 108 h, followed by ethanol fermentation at 30 °C. The hydrolysis was started with 7% APCS uploading, and four feedings were carried out at 12, 24, 36, and 48 h, respectively, each with 4, 4, 4, and 1% solid uploading. Conditions for SSF were almost like SHF except that the fermentation temperature was maintained at 37 °C throughout the process and commercial yeast was inoculated at 12 h in SSF.

### Analytical methods

Total reducing sugars, filter paper activity (FPA), BGA, and xylanase activities were assayed according to the methods described elsewhere [20–22]. The cellulosic composition of both corn stover and APCS were determined via the analytical procedures recommended by NREL. The concentration of glucose and ethanol was detected by HPLC (Waters 410, Waters, MA) equipped with an Aminex HPX-87H column (300 mm × 7.8 mm, Bio-Rad, Hercules, CA). The column oven temperature was set at 65 °C. Samples were eluted with 0.004 M H_2_SO_4_ at a flow rate of 0.6 mL/min. The glucose and ethanol yields were calculated as follows:

$${\text{Glucose yield (\%)}} = \frac{{{\text{Glucose }}\left( {\text{g}} \right) \times 0.9}}{{{\text{Cellulose in substrate }}\left( {\text{g}} \right)}} \times 100\%$$

$${\text{Ethanol yield (\%)}} = \frac{{{\text{Ethanol }}\left( {\text{g}} \right)}}{{{\text{Glucose in substrate }}\left( {\text{g}} \right) \times 0.51}} \times 100\%$$

All results are presented as the average values of three independent experiments with standard deviations at significance *p* < 0.05.

## Results and discussion

### Cellulase overproduction by fed-batch culture

**Fig. 1:**
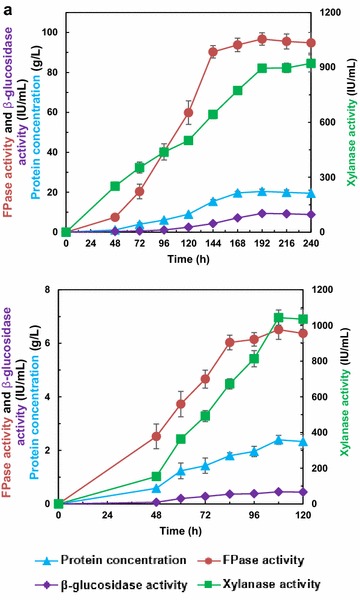
Fed-batch culture of *T. reesei* Rut C30 for cellulase production with MGD (**a**) or APCS (**b**) as inducer

Fed-batch fermentation strategy was employed for *T. reesei* cellulase production using MGD or APCS as inducer. The fermentation process consists of two steps, namely, rapid growth process in the excess of the substrate, followed by cellulase production phase performed in fed-batch mode under carbon limitation [23]. Figure 1 illustrates the time course of the fed-batch culture of *T. reesei* Rut C30. The results of MGD (Fig. 1a) were in accordance with our previous study [6]. It was of interest to see that cellulase yield and productivity of MGD (144 h) were 13.87 and 10.40 times, respectively, higher than that of APCS at 108 h (Fig. 1b). Obviously, MGD presents great advantage for cellulase production in *T. reesei*, and the characteristics of the enzymatic cocktail were further studied.

The two types of crude enzymes Cel-MGD and Cel-CS achieved from MGD to APCS feeding strategies showed great difference in cellulase, β-glucosidase and xylanase activities (Table 1). The potential of the two enzymes for biomass degradation was further evaluated. Under the same protein concentration, the specific activities of cellulase and β-glucosidase of Cel-MGD were 0.74 and 1.44 times higher than that of Cel-CS, respectively, which exhibit the robust ability in cellulose degradation. However, the hemicellulose conversion would be more favorable in Cel-CS due to the higher specific xylanase activity (9.95 times) than that of Cel-MGD.

**Table 1:** Effect of different inducers on cellulolytic enzyme activities

Enzyme	Protein (mg/mL)	Specific activity (IU/mg protein)		
		FPase	Cellobiase	Xylanase
Cel-CS	2.39	2.72	0.19	436.64
Cel-MGD	20.41	4.74	0.46	43.88

### Hydrolysis efficiency of cellulases cocktail in *T. reesei* Rut C30

**Fig. 2:**
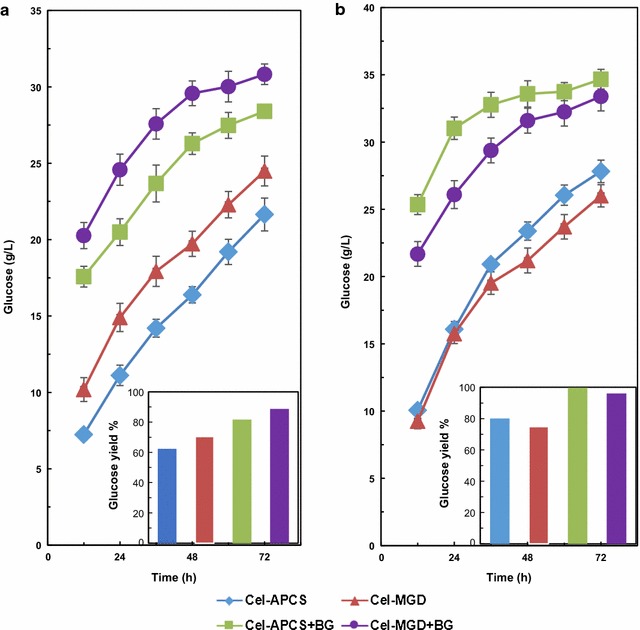
Time course of the enzymatic hydrolysis for glucose concentration and yield. **a** 3 mg protein/g APCS; **b** 20 FPU/g APCS

The saccharification ability of Cel-MGD and Cel-CS was further examined during time course hydrolysis of APCS. Interestingly, opposite results were obtained when the crude enzymes were evaluated using the same protein (3 mg/g APCS) or the same FPA (20 FPU/g APCS) loading under the same reaction conditions (Fig. 2). When 3 mg protein/g APCS was employed (Fig. 2a), 24.49 g/L glucose (70.46% glucose yield) was released using Cel-MGD, which was higher than that of 21.65 g/L glucose (62.27% yield) from Cel-CS. The results were supported by the results of enzymatic activities (Table 1), indicating the effectiveness of Cel-MGD for APCS hydrolysis. Surprisingly, the glucose concentration (27.82 g/L) produced by 20 FPU Cel-CS/g APCS was higher than that released by Cel-MGD (26.01 g/L) (Fig. 2b). The same FPA meant the same amount of cellulase, ignoring the xylanase and other glycoside hydrolases in cellulolytic cocktails. Cellulase produced by *T. reesei* comprises three major enzyme components: cellobiohydrolases, endoglucanases, and β-glucosidases [8], along with some other elements involved in cellulose degradation, for instance, the expansin-like protein swollenin (SWO1) and GH61 polysaccharide monooxygenases (PMOs), which have been shown to enhance lignocellulose degradation [9]. Therefore, different components in cellulases may be the reason for the above results. Aiming to further optimize the crude enzymes induced by MGD, the lignocellulolytic enzyme profiles of Cel-MGD and Cel-CS were further compared by secretome analysis, and described in the below section.

The β-glucosidase (BG) activity is low in cellulase cocktails by *T. reesei*, which leads to disaccharide accumulation, resulting in a reduced cellulose degradation efficiency [12–14, 24]. It was interesting to find that higher BGA was obtained in Cel-MGD than Cel-CS. Extra BG (FPA:BGA = 1:2) was added into Cel-MGD and Cel-CS to evaluate whether enough BG is present in the crude enzymes (Fig. 2). It was found that the released glucose was improved 25.84% using Cel-MGD + BG compared with the crude enzyme, indicating that the *T. reesei* cellulase using MGD as inducer was still weak in BGA.

### Different protein profiling using two inducers by analysis of *T. reesei* secretome

More glucose was released in APCS hydrolysis by Cel-CS than by Cel-MGD when the same FPA was used for evaluation (Fig. 2b), which means different hydrolysis efficiency of the crude enzymes using APCS and filter paper (FP) as substrates. The biomass hydrolysis rate is one of the major limitations towards the commercialization of second-generation biofuel, which contained various rate-limiting factors (enzyme adsorption, crystallinity, accessibility, reactivity, enzyme clogging, and degree of polymerization) [25]. In this study we focused on enzyme reactivity by optimizing enzyme compositions.

The iTRAQ system was employed for quantitative analysis of the secretome of *T. reesei* Rut C30 induced by MGD or APCS. In total, 242 proteins were identified using Maxquant after applying the cutoff of unused protein score > 2, which corresponds to a 99% confidence level and a false discovery rate < 1.0%. The proteins identified were sorted with N-terminal Sec-dependent secretion signal using SignalP 3.0 and classified according to the biological functions. A total of 143 secreted proteins with signal peptides were identified, which were sorted into cellulases, hemicellulases, peptides, chitinase, phosphatases, transport, and hypothetical proteins. Other proteins without any predicted signal peptides were probably detected due to non-conventional mechanisms of secretion, or by possible cell lysis and/or death, which was also proposed in the previous studies [26, 27]. Interestingly, there are similar components for exocellular proteins induced by the MGD and APCS, in which 57.3% proteins were putative glycoside hydrolases (78 proteins) and esterases (4 proteins) that are related to lignocellulosic biomass degradation. In the previous secretome analysis of *T. reesei* Rut C30 during solid fermentation, similar amounts to secreted proteins and lignocellulose degradation-related proteins were identified (135 and 68, respectively) [27]. In another secretome study of *T. reesei* Rut C30 grown on lignocellulosic biomass (solid-state culture), 84 glycosyl hydrolases were identified by iTRAQ [26]. However, the protein diversity detected in this study is not comparable with the previous studies [26, 27], which may be due to different culture mode. All the identified proteins are listed in Additional file 2: Table S2. Fold change (FC) of main cellulosic enzymes' abundance differences detected common to the Cel-MGD and Cel-CS are listed in Table 2.

**Table 2:** Fold change of protein abundance differences detected for main cellulosic enzymes in *Trichoderma reesei* Rut C30 common to MGD and APCS as inducer

Category	Protein ID	Name	FC MGD/APCS
Cellulase: main activity	125125	Exoglucanase 1	1.67
	122470	Exoglucanase 2	1.85
	5304	Endoglucanase 1	2.59
	72489	Endoglucanase 2	1.20
	136547	β-Glucosidase 1	1.38
Cellulase: accessory proteins	139633	Endoglucanase 4 (AA9)	0.36
	122518	Endoglucanase 7 (AA9)	0.70
	104220	Swollenin	0.64
	121449	Cellulose binding domain CIP1	0.78
	125575	Cellulose binding domain CIP2	0.65
Hemicellulase: main chain activities	140746	β-Xylosidase	0.28
	12549	β-Mannosidase	0.50
	124931	Endoxylanase	0.39
	38418	Endo-1,4-beta-xylanase	0.49
	111943	Xyloglucanase	0.40
Hemicellulase: side chain activities	6433	α-d-Galactosidase	0.43
	101346	β-Galactosidase	0.48
	12566	α-Galactosidase 2	0.35
	102517	α-l-Arabinofuranosidase B	0.33
	72252	α-l-Arabinofuranosidase B	0.52
	118070	α-*N*-Arabinofuranosidase 2	0.51
	90302	α-Glucuronidase	0.39
	136770	Acetyl esterase	1.71
	139631	Acetylxylan esterase	0.61
Pectinase	11580	Endo-beta-1,6-galactanase	0.68
	133383	Endo polygalacturonase	0.34
Chitinase	99285	β-*N*-Acetylhexosaminidase	3.10

*FC* fold change

The same components of the extracellular proteins from *T. reesei* Rut-C30 induced by two different inducers (MGD and APCS) may be due to the presence of wheat bran in the culture medium, which contains cellulose and was supplemented as nutrients. However, our recent study [28] suggests that APCS induced higher xylanase activities, which is consistent with the current secretome data (Table 2), and also with much higher xylanase activity we detected in Cel-CS (Table 1). Significant differences in the ratio of cellulases and hemicellulases were revealed. Higher quantities of two cellobiohydrolases CBH1, CBH2, as well as EG2, EG3, and EG5 were detected in Cel-MGD. In addition, there are four β-glucanases (BGL1, BGL2, and CEL3j and CEL3e) detected by iTRAQ analysis, and the ratios (MGS/CS) for iTRAQ results are 1.38, 1.71, 2.59, and 2.59, respectively. The higher amount of these proteins is consistent with the higher FPase and BG activities in Cel-MGD as shown in Table 1. It is well-known that multiple enzymes must work together to degrade cellulose into glucose. Therefore, Cel-MGD showed stronger cellulose degradation ability than Cel-CS under the same protein dosage (Fig. 2a). Moreover, degradation of lignocellulose demands more accessory proteins such as SWO1, glucuronoyl esterase (CIP1 and CIP2), and AA9 lytic polysaccharide monooxygenases (LPMOs) [9, 29]. In consistence with more glucose release by Cel-CS when the same FPA was used, there were more auxiliary proteins in Cel-CS, including two LPMOs (CEL61a and CEL61b), which were 2.81 and 1.43 times higher in Cel-CS than that in Cel-MGD, respectively. In addition, SWO1, CIP1, and CIP2 contents reached about 3.36, 1.55, and 1.56 folds in Cel-CS to that in Cel-MGD. LPMOs are capable of oxidizing recalcitrant polysaccharides, which showed a synergistic effect with lignocellulose on degradation of a range of cellulosic substrates [9, 30]. Therefore, Cel-CS was more effective on APCS hydrolysis than Cel-MGD under the same conditions of FP degradation. Other glycoside hydrolases in Cel-MGD are significantly lower than that in Cel-CS, such as GH2 (candidate β-mannosidase), GH16 (candidate glucan endo-1,3(4)-β-D-glucosidase), GH17 s (candidate glucan endo-1,3-β-glucosidases), GH30 (candidate endo-β-1 6-galactanase), GH36 (α-galactosidase), GH71 (candidate α-1,3-glucanase), GH76 (candidate α-1,6-mannanase), GH79 (candidate β-glucuronidase), GH92 (candidate α-1,2-mannosidase), and chitinase (Additional file 2: Table S2). Induction of cellulase production in *T. reesei* by the mixture of MGD and APCS was tested in our previous work, and better degradation effect was observed when optimized ratio of MGD and APCS was employed [28]. The data of secretory proteins in this study further revealed new players in cellulose degradation, especially auxiliary proteins secreted in response to APCS which are in low abundance when MGD was employed as inducer. We have tested the effects of overexpression of *swo1*, *cip1*, and *cip2* in *T. reesei* Rut C30 using the *eno1* promoter, but no improvement of cellulase production was observed (data not shown). Addition of accessory proteins may be an alternative way. It was reported that adding small amount of lytic polysaccharide monooxygenase AA9 (formerly named GH61) as an accessory enzyme enhanced hydrolysis of pretreated polar and corn stover [31]. In another study, supplementation of pectinase and α-l-arabinofuranosidase increased hydrolysis efficacy of pretreated sugarcane bagasse [32]. These students emphasized the importance of optimization of enzyme compositions for efficient lignocellulose degradation.

### Impact of over-expressing β-glucosidase in *T. reesei*

Although high cellulase titer was obtained using MGD as inducer, BGA was still not sufficient for efficient lignocellulose hydrolysis. Activity of β-glucosidases in *T. reesei* is normally low, and therefore commercial β-glucosidase is required for efficient biomass hydrolysis [6]. Synergistic effects of some β-glucosidases and *T. reesei* cellulase were also reported [33]. Genetic engineering has been successfully applied to increase BGA, but in the all reported cases, cellulose-inducible promoters were used, and the disadvantage of such promoters is the reduced expression of cellulase or xylanase genes [12–14, 34, 35]. It was reported that *pdc1* (encoding pyruvate decarboxylase) in *T. reesei* Rut C30 was expressed at high level and *pdc1* promoter could constitutively promote heterologous gene expression using glucose as the sole carbon source [36, 37], it is therefore suitable to use *pdc1* promoter to drive the expression of target genes by MGD induction owing to abundant glucose exist in MGD [6].

The *aabgl1* encoding β-glucosidase 1 (*aabgl1*) from *A. aculeatus* with high specific activity was used as the target gene for achieving higher BGA in *T. reesei* [13]. The plasmid pCBHYGB-PB containing *aabgl1* expression cassette was constructed (Additional file 3: Figure S1A), which was then transformed into the parent strain *T. reesei* Rut C30. Twenty recombinants with genetic stability were selected and verified for *aabgl1* cassette by PCR. All of the selected transformants (Additional file 3: Figure S1B) possessed the BGA of 0.44–3.96 IU/mL by 10 g/L MGD as the inducer in shake flask culture. The highest BGA (3.96 CBU/mL) of the recombinant strains was 26.4 times of that of the control (0.15 CBU/mL). These results demonstrated that the heterologous β-glucosidase gene was efficiently expressed using MGD as a carbon source and the enzyme was continuously secreted into the fermentation broth.

The large difference of BGA among the mutants might be due to the different expression levels of *aabgl1*. The copy number of *aabgl1* gene in the recombinants was determined, which is one of the factors affecting the transcription [14]. Four mutants with large improvement in BGA were selected for copy number analysis using qPCR, and the results showed that the transformants *T. reesei* PB-3 and PB-4 had four and three copies, respectively, while both PB-14 and PB-17 had two copies of *aabgl1* gene in the genome (Additional file 4: Figure S2). High copy numbers integrated into the genome was consistent with higher expression levels of the heterologous gene. However, *T. reesei* PB-14 and PB-17 harboring the same copies of *aabgl1* have displayed different BGA, which illustrated that the integration locus also affects the expression of the heterologous gene. Previous studies also demonstrated that integration sites play important roles in the elevation of β-glucosidase activity in the recombinant *T. reesei* strains [38, 39]. In our previous studies, integration of the new regulatory gene *Trvib*-*1* in a putative strong integration site resulted in higher enhancement of cellulase production [40]. Another possibility is the position effect which was reported in budding yeast *Saccharomyces cerevisiae* [41]. However, systematic survey of the effect of integration sites on gene expression levels in *T. reesei* has still not been performed, which will be an interesting topic for future work.

**Fig. 3:**
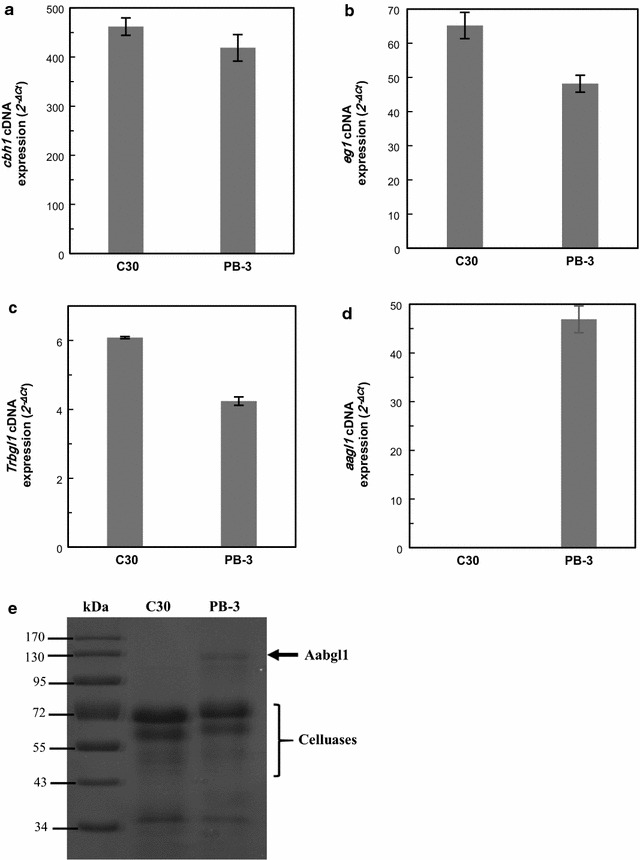
Transcription of gene encoding cellulase (**a** *cbh1*; **b** *eg1*; **c** *Trbgl1*; and **d** *aabgl1*). The value is the mean of three independent experiments with standard deviation, which was calibrated by the 2^−ΔCt^ method. **e** SDS-PAGE analysis of Aabgl1 expression of recombinant *T. reesei* PB-3 and the parent strain *T. reesei* Rut C30

The recombinant strain *T. reesei* PB-3 with the highest BGA was selected and investigated further. The expression levels of major cellulase genes (*cbh1*, *eg1*, *Trbgl1*, and *aabgl1*) in *T. reesei* Rut C30 and PB-3 were detected using 10 g/L MGD in shake flask culture for 36 h by qPCR analysis (Fig. 3a–d). Moreover, there was a distinct band at about 130 kDa in *T. reesei* PB-3 through SDS-PAGE analysis which is absent in *T. reesei* Rut C30, further confirming the expression of AaBG1 (Fig. 3f). The relative *aabgl1* expression level was shown to be about 11% of the *cbh1* expression level in *T. reesei* PB-3, indicating that *pdc1* promoter could promote gene transcription efficiently in MGD. However, the expression levels of *cbh1*, *eg1*, and *Trbgl1* in *T. reesei* PB-3 were lower than the parent strain *T. reesei* Rut C30. The possible reason was that higher BGA in the recombinant strain *T. reesei* PB-3 (26.4 times than that of *T. reesei* Rut C30) would hydrolyze β-disaccharides into glucose, which resulted in lower induction of cellulose. These results implied that BGA must be controlled at proper level to avoid significant reduction in cellulase gene expression. Until now, studies on promoters of *T. reesei* are still limited [42]. It will be ideal if the promote activity used to overexpress β-glucosidase was activated at latter fermentation stages after induction of cellulase.

### Cellulase and β-glucosidase overproduction by fed-batch culture

**Fig. 4:**
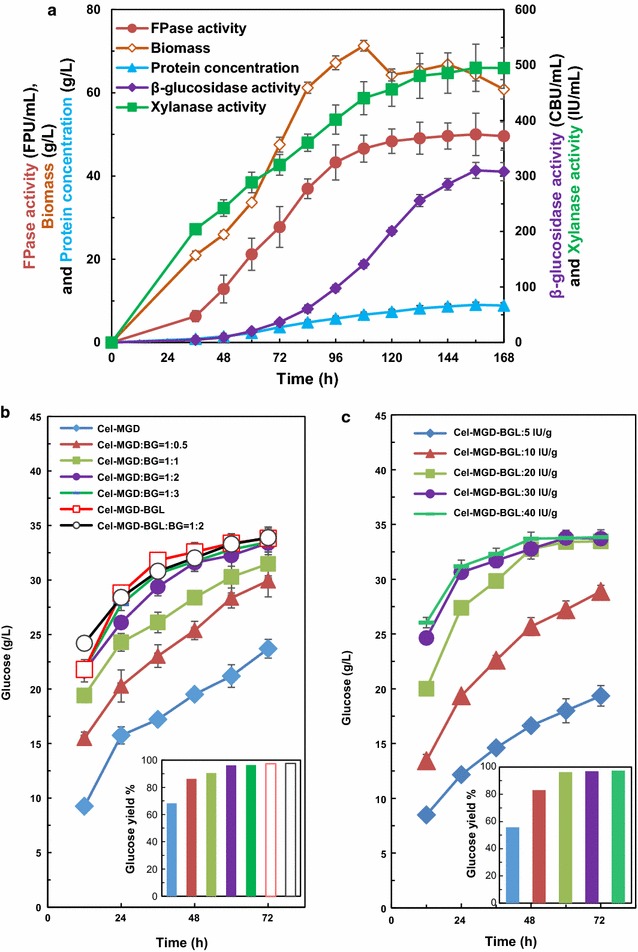
Fed-batch culture of *T. reesei* PB-3 with MGD as inducer for cellulase production (**a**) and saccharification of APCS by crude cellulase from host strain *T. reesei* Rut C30 or recombinant *T. reesei* PB-3 (**b**, **c**). **a** The time course of *T. reesei* PB-3 using MGD as inducer; **b** Saccharification of APCS by Cel-MGD (produced by *T. reesei* Rut C30) and Cel-MGD-BGL (produced by *T. reesei* PB-3) with/without commercial β-glucosidase for a ratio of enzyme activity ranging from 1:0.5 to 1:3. **c** Saccharification of APCS by Cel-MGD-BGL with different cellulase loading

The fed-batch culture was performed for achieving a higher activity of β-glucosidase and cellulase. Figure 4a illustrated the time course of *T. reesei* PB-3 using MGD as substrate and inducer. BGA was enhanced significantly to 310.1 CBU/mL at 156 h due to the overexpression of the heterologous β-glucosidase, which was more than 71 folds of that in the host strain *T. reesei* Rut C30. As a result, the maximum β-glucosidase productivity reached 1988.0 CBU/h/L. The cellulase and xylanase activities and extracellular protein secretion of PB-3 were lower than *T. reesei* Rut C30 because inducible β-disaccharides were degraded with the accumulation of β-glucosidase [6]. However, the cellulase activity was still as high as 50.0 FPU/mL, which was more than 2.94 and 2.2 times of that obtained using cellulose [43] and lactose [44] as inducer, respectively. The high cellulase and β-glucosidase activities obtained simultaneously in *T. reesei* PB-3 would benefit in situ use of the crude enzymes without concentration for achieving high titer of bioethanol, which would help to reduce the cost involved in product separation.

Extensive studies have been focused on the overexpression of heterologous β-glucosidase in *T. reesei*, which always employed cellulase or xylanase promoter with cellulose as an inducer, and reduced expression of corresponding genes [12–14]. Furthermore, these recombinant strains have not been examined in submerged fermentation through fed-batch culture for β-glucosidase production. *Aspergillus* sp. has been used in industry by solid-state fermentation. However, *Aspergillus* sp. contamination associated with the solid-state culture makes the cellulase not suitable for hydrolysis of lignocellulose for biofuels and bio-based chemical production [45–47]. On the other hand, the β-glucosidase titer was still low by *Aspergillus* sp. in submerged fermentation, which showed only 9.3 IU/mL and productivity of 1.78 U/mL/day [48]. Therefore, submerged culture of *T. reesei* PB-3 for efficient simultaneous β-glucosidase and cellulase production at large scale has considerable potential for bioconversion of agricultural residues.

To evaluate the hydrolysis ability of the cellulase cocktails of *T. reesei* Rut C30 or PB-3 on natural cellulosic materials, the crude enzyme from *T. reesei* Rut C30 (Cel-MGD) or PB-3 (Cel-MGD-BGL) were used to hydrolyze APCS. Cel-MGD was found to be lack of BGA in biomass hydrolysis [6]; although Cel-MGD-BGL had higher BGA, additional β-glucosidase (BG) was also supplemented to examine whether the self-BG was sufficient in this study. Figure 4b showed that 33.82 g/L glucose was released using Cel-MGD-BGL at 72 h with a glucose yield of 97.29%, meanwhile, 33.88 g/L glucose was generated by Cel-MGD-BGL + BG, indicating that the crude Cel-MGD-BGL was efficient for biomass degradation and commercial BG was not required. In addition, the highest amounts of released glucose were 33.47 and 33.40 g/L from Cel-MGD: BG = 1: 3 and Cel-MGD: BG = 1: 2, respectively, but only 23.70 g/L glucose was obtained without additional BG. Moreover, the glucose yield was lower when Cel-MGD: BG < 1:2. More importantly, Cel-MGD-BGL was able to hydrolyze APCS efficiently without extra BG, which was well-matched with Cel-MGD + BG. The effect of the Cel-MGD-BGL loading on hydrolysis performance was investigated at five different dosages (5, 10, 20, 30, and 40 FPU/g APCS). Within 72 h, glucose was completely released under cellulase loading of 20, 30, and 40 FPU/g, but glucose concentration was lower at 20 than that of 30 and 40 FPU/g before 48 h. When the enzymes loading was decreased to 5 and 10 FPU/g, glucose concentration was reduced to 42.8 and 14.63%, respectively, compared to that of 30 FPU/g at 48 or 72 h (Fig. 4c). In summary, the cellulase loading at 30 FPU/g was optimum for cellulosic ethanol production.

### Bioethanol production from corn stover using in-house produced *T. reesei* enzymes

In our previous study, 44.8 g/L ethanol was produced from APCS by Cel-MGD + BG [6]. However, it is important to achieve higher concentration of glucose from APCS to increase product concentration and reduce purification costs [49–51]. Therefore, fed-batch strategy was adopted in this study for corn stover hydrolysis with a total biomass uploading of 20% by SHF or SSF process.

**Fig. 5:**
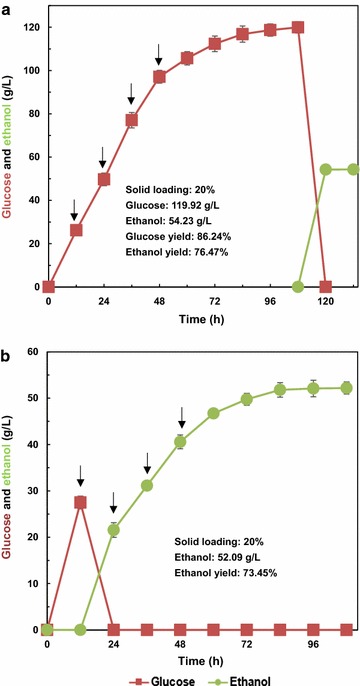
Fed-batch saccharification and ethanol fermentation with a total uploading of APCS at 20%. The crude cellulase from recombinant *T. reesei* PB-3 was supplemented at 30 FPU/g DM. Arrows indicate the time for biomass feeding. **a** SHF process and **b** SSF process

Figure 5a shows that the glucose was generated consistently during the process of saccharification, and the glucose concentration reached 119.92 g/L at 108 h with the yield of 86.24% by SHF, indicating that APCS could be hydrolyzed efficiently by the crude cellulase Cel-MGD-BGL. Once the *S. cerevisiae* was added into the fermenter, glucose was metabolized rapidly within 12 h, and the ethanol concentration reached 54.23 g/L with the ethanol yield of 76.47%. When SSF process was applied for bioethanol production, 52.09 g/L ethanol was produced within 96 h and the ethanol yield reached 73.45% (Fig. 5b). Although the ethanol yield was lower by SSF than SHF, the productivity reached 0.54 g/L/h using SSF, which was higher than 0.45 g/L/h in the case of SHF. Compared with SHF, SSF encompasses the hydrolysis and fermentation process within the same vessel, which was more favorable for bioethanol production because of the shorter processing time, lower cost of equipment, and lower contamination risk [52]. However, higher sugar conversions and higher ethanol yield were achieved by SHF in this study because low temperature (37 °C) was not suitable for saccharification of Cel-MGD-BGL.

Comparison of cellulosic ethanol and productivity from corn stover obtained in this study using in-house cellulase with those reports by other commercial cellulase is shown in Table 3 [53–55]. The results indicated that in-house cellulase production by the recombinant *T. reesei* PB-3 was effective to replace commercial enzyme for biomass saccharification. Besides, effective bioconversion for glucose was also achieved in this work. The results suggested that *T. reesei* PB-3 has great potential for efficient bioconversion of cellulosic biomass.

**Table 3:** Comparison of cellulosic ethanol production from pretreated corn stover

Fermentation mode	Solid loading (%, w/w)	Ethanol titer (g/L)	Ethanol yield	References
SSF	30	59.1	75.9%	[53]
SSF	25	55.7	79.5%	[54]
SSCF	25	47.2	65.5%	[55]
SHF	20	54.2	76.47%	This study
SSF	20	52.1	73.45%	This study

*SSF* simultaneous saccharification and fermentation; *SHF* separate hydrolysis and fermentation; *SSCF* simultaneous saccharification and co-fermentation

## Conclusions

The secretome of *T. reesei* RUT C30 with MGD or APCS as inducer was compared, which indicated that cellulolytic enzymes induced by MGD had higher content of major cellulases, but lower BGA still remains a challenge. To further improve the hydrolysis efficiency of the cellulytic enzyme cocktail, *T. reesei* PB-3 was developed through overexpression of *A. aculeatus* β-glucosidase gene under a constitutive *pdc1* promoter, which substantially improved β-glucosidase production. Furthermore, overproduction of cellulases and β-glucosidase was achieved simultaneously by *T. reesei* PB3 through the fed-batch fermentation using MGD as the inducer. The crude enzyme was used for the hydrolysis of APCS, and bioethanol fermentation was achieved through SHF and SSF, respectively. To our knowledge, this is the first report that high activities of cellulases and β-glucosidase were achieved simultaneously for lignocellulosic bioconversion, and the crude enzyme can be used for cellulosic hydrolysis and ethanol production more efficiently.

## Additional files

**Additional file 1: Table S1.** Primers used for vector construction, qPCR and determination of copy numbers.

**Additional file 2: Table S2.** Identified secreted proteins induced by MGD and CS.

**Additional file 3: Figure S1.** Construction of the *aabgl1* overexpression *T. reesei* strains. A: Map of *aabgl1* expression vector pCBHYGB-PB; B: Comparison of β-glucosidase production of the recombinant transformants and the host strain *T. reesei* Rut-C30; C: Detection of *aabgl1* copy numbers in the transformants by qPCR analysis. *Tef1α* gene was used as a single copy control.

**Additional file 4: Figure S2.** Detection of *aabgl1* copy numbers in the transformants by qPCR analysis. *Tef1α* gene was used as a single copy control.

## Abbreviations

FPU: filter paper unit
CBU: cellobiase unit
MGD: mixture of glucose and β-disaccharides
APCS: alkali pretreatment corn stover
qPCR: quantificational real-time polymerase chain reaction
iTRAQ: isobaric tags for relative and absolute quantification
SDS-PAGE: SDS-polyacrylamide gel electrophoresis
Cel-CS: enzymatic cocktails were produced using APCS as inducer
Cel-MGD: enzymatic cocktails produced with MGD as inducer
