# Supplementary material for: Optimization of cellulolytic enzyme components through engineering Trichoderma reesei and on-site fermentation using the soluble inducer for cellulosic ethanol production from corn stover
Source: Biotechnol Biofuels. 2018 Feb 23;11:49. doi: 10.1186/s13068-018-1048-5 (PMC5824536; doi:10.1186/s13068-018-1048-5)
Supplement: Supplementary file 1 — Additional file 1: Table S1. Primers used for vector construction, qPCR and determination of copy numbers. [file 13068_2018_1048_MOESM1_ESM.docx]

**Additional data for**

**Optimization of cellulolytic enzyme components through engineering *Trichoderma reesei* and on-site fermentation using the soluble inducer for cellulosic ethanol production from corn stover**

Yong-Hao Li^1#^, Xiao-Yue Zhang^2^, Fei Zhang^1^, Liang-Cai Peng^1^, Da-Bing Zhang^1^, Akihiko Kondo^3^, Feng-Wu Bai^1^, Xin-Qing Zhao^1*^

^1^State Key Laboratory of Microbial Metabolism, Joint International Research Laboratory of Metabolic and Developmental Sciences, and School of Life Science and Biotechnology, Shanghai Jiao Tong University, Shanghai, 200240, China

^2^School of Life Science and Biotechnology, Dalian University of Technology, Dalian 116023, China.

^3^Department of Chemical Science and Engineering, Graduate School of Engineering, Kobe University, Kobe 657-8501, Japan.

^#^Present address: School of Chemistry and Chemical Engineering, Chongqing University of Science and Technology, Chongqing 401331, China.

**Correspondence:** Prof. Xinqing Zhao, School of Life Science and Biotechnology, Shanghai Jiao Tong University, Shanghai 200240, China.

**E-mail**: [xqzhao@sjtu.edu.cn](mailto:xqzhao@sjtu.edu.cn).

**Appendix A. Additional data**

**Additional table**

Table S1. Primers used for vector construction, qRT-PCR and determination of copy numbers^*^

| Primer | | | Sequence |
| --- | --- | --- | --- |
| Primers for plasmid construction | | | |
| hygB | -F | 5’-GGACTAGTCCTTGTATCTCTACACACAGGCTCA-3’ | |
|  | -R | 5’-GGACTAGTCCTTAATTAATCGAGTGGAGATGTGGAGTGGGCGC-3’ | |
| Ppdc | -F | 5’-CCCCCCTCGAGGTCGACGGTAGGACTTCCAGGGCTACTTG-3’ | |
|  | -R | 5’-TCAAGCCAACTGAGCTTCATGATTGTGCTGTAGCTGCGCT-3’ | |
| Tpdc | -F | 5’-CGCTCCCGAAGGTGCAATGACCCGGCATGAAGTCTGACCG-3’ | |
|  | -R | 5’-AGGAATTCGATATCAAGCTTTGGACGCCTCGATGTCTTCC-3’ | |
| aabgl1 | -F | 5’-AGCGCAGCTACAGCACAATCATGAAGCTCAGTTGGCTTGA-3’ | |
|  | -R | 5’-CGGTCAGACTTCATGCCGGGTCATTGCACCTTCGGGAGCG-3’ | |
| Primers for qRT-PCR analysis | | | |
| *sar1* | -F | 5’-CGTCTTGTCGTCTTTGGGTCT-3’ | |
|  | -R | 5’-GGATAGCAACTCGGTCGTTCTT-3’ | |
| *cbh1* | -F | 5’-ACGAGTTCTCTTTCGATGTTGATG-3’ | |
|  | -R | 5’-CGGTGTTGGTGGGATACTTG-3’ | |
| *eg1* | -F | 5’-CTGGTGGCTAGTGTTGAGGG-3’ | |
|  | -R | 5’-CCGAGTGATCTGTTCCAGAATGT-3’ | |
| *Trbgl1* | -F | 5’-CCGAGTGATCTGTTCCAGAATGT-3’ | |
|  | -R | 5’-CTGGGTGCTGAAGATGGGTAG-3’ | |
| *aabgl1* | -F | 5’-GGTCAGGCTATGGGTCAAGA-3’ | |
|  | -R | 5’-CGCAAAGAGCACACCAGTAA-3’ | |
| Primers for copy numbers analysis | | | |
| *tef1α* | -F | 5’-ACCAAGGCTGGCAAGTTC-3’ | |
|  | -R | 5’-GACACCAGTCTCGATACG-3’ | |
| *aabgl1* | -F | 5’-GGTCAGGCTATGGGTCAAGA-3’ | |
|  | -R | 5’-CGCAAAGAGCACACCAGTAA-3’ | |

^*^The underlines are the site of the restriction enzyme.
