# Supplementary material for: Optimization of cellulolytic enzyme components through engineering Trichoderma reesei and on-site fermentation using the soluble inducer for cellulosic ethanol production from corn stover
Source: Biotechnol Biofuels. 2018 Feb 23;11:49. doi: 10.1186/s13068-018-1048-5 (PMC5824536; doi:10.1186/s13068-018-1048-5)
Supplement: Supplementary file 4 — Additional file 4: Figure S2. Detection of aabgl1 copy numbers in the transformants by qPCR analysis. Tef1α gene was used as a single copy control. [file 13068_2018_1048_MOESM4_ESM.docx]

**Additional data for**

**Optimization of cellulolytic enzyme components through engineering *Trichoderma reesei* and on-site fermentation using the soluble inducer for cellulosic ethanol production from corn stover**

Yong-Hao Li^1#^, Xiao-Yue Zhang^2^, Fei Zhang^1^, Liang-Cai Peng^1^, Da-Bing Zhang^1^, Akihiko Kondo^3^, Feng-Wu Bai^1^, Xin-Qing Zhao^1*^

^1^State Key Laboratory of Microbial Metabolism, Joint International Research Laboratory of Metabolic and Developmental Sciences, and School of Life Science and Biotechnology, Shanghai Jiao Tong University, Shanghai, 200240, China

^2^School of Life Science and Biotechnology, Dalian University of Technology, Dalian 116023, China.

^3^Department of Chemical Science and Engineering, Graduate School of Engineering, Kobe University, Kobe 657-8501, Japan.

^#^Present address: School of Chemistry and Chemical Engineering, Chongqing University of Science and Technology, Chongqing 401331, China.

**Correspondence:** Prof. Xinqing Zhao, School of Life Science and Biotechnology, Shanghai Jiao Tong University, Shanghai 200240, China.

**E-mail**: [xqzhao@sjtu.edu.cn](mailto:xqzhao@sjtu.edu.cn).

Fig. S2. Detection of *aabgl1* copy numbers in the transformants by qRT-PCR analysis. *Tef1α* gene was used as the single copy control.

Figure S2
